# "As Age Creeps On": Possible Troubles and How to Treat Them

**Published:** 1917-12-08

**Authors:** 


### "AS AGE CREEPS ON."

### Possible Troubles and How to Treat Them.

In a letter under date November 1885, the late Sir William Gull wrote: "The evils of life are much balanced. Old man prostate trouble; woman prolapsus uteri and so on." The time which has elapsed since this letter has seen much development in the treatment of both of these conditions and has helped many to live and be thankful. But years of war, anxiety, and depression have recently brought under notice the slighter degrees of these states by making less easily borne the symptoms which depend on them. It is undoubtedly true that the vast majority of cases of prostatic enlargement which call for operative treatment have a distant history of gonococcal infection. With such a causation the removal of the focus of infection is the logical treatment. But we must keep in mind the part which the prostatic secretion plays in stimulating the agility of the spermatozoa, and recognise that the function of the gland is likely to be more severely taxed in later than in earlier life; also that a limited increase in size, even with some fibroid degeneration, hardly oversteps the limits of physiological change. Similarly, fibrous changes take place in the vagina, after the menopause, and serve the purpose of aiding the uterus to resist prolapse—one of the age penalties to which Sir William Gull refers. These adhesions do not prevent retroversion of the uterus, which may be found in a position pressing as much back upon the rectum as the prostate of the male. There is this difference, that while the vaginal bands follow the menopause, the prostatic enlargement precedes the end of sexual power by at least ten years. The most early result of prostatic growth is the formation of a shallow sulcus in the bladder, behind the swollen gland, which becomes the resting-place for a small quantity of urine at the end of micturition. It is true that in a normal bladder this need not mean the occurrence of decomposition; but some kinds of food and drink render the urine irritating; some emotional influences increase the frequency of micturition, and the close relation of the base of the bladder to the intestine makes the pilgrimage of the *Bacillus coli* easy. Experience tells us that such infection is not always lasting, as is the infection of the gonococcus, but the congestion produced inevitably leaves the prostate a little larger. With such an increase the gerontal constipation, upon which Dr. Hollis wrote a classical paper a few years since, begins, and the serenity of life is disturbed (*British Medical Journal*, vol. i., 1916, page 679). The war bread, with its increased and harder residuum in the fæces, favours a stasis near the sigmoid flexure and in the upper rectum. The result is, too often, an incomplete emptying of both bladder and bowel and an increased frequency of calls. Physiological guidance is worthy of a trial before resort to drugs. In early and middle life men are apt to neglect the time-table of micturition. The first indication of danger is a little hesitation in starting the act, which produces the sensation of alarm, followed by a spasmodic rush, which expends itself before the bladder is quite empty. A quiet mind, and a slight effort to hold back the flow of urine will often effect a start; and so soon as the act has begun the repetition of a column of the multiplication table or a verse of poetry which is not too familiar, will permit the bladder to complete its work and to rest quietly until the next hour on the time-table. It is said that at sixty years of age a man begins to micturate once during his sleeping hours, and that an additional nightly call is added for each of the three or four succeeding years. The time-table with the aid of the procedure mentioned breaks this rule, often to a wonderful extent, and there are men in the late 'seventies who sleep quietly through the night with only one disturbance, or even without a call. Some accessories are needful. On rising micturate and drink a glass of boiled water. At breakfast not more than one cup of tea or coffee should be taken, to secure a rest of the bladder until luncheon-time. It is well known that the bladder holds a more concentrated urine better than a more limpid fluid. Little fluid, or better still none, should be taken with luncheon, but a full supply with afternoon tea, for this may be carried comfortably until dinner-time. Little fluid should be taken with dinner, and one tumblerful or a little more an hour before bed-time should close the day and secure starting the night with a good prospect of undisturbed rest. Alcohol disturbs the night unless it be taken in very small quantity and largely diluted—not more than one tablespoonful to a tumbler of water. In this quantity and dilution it does not irritate a normal bladder.

Those who are growing old and who belong to the uncircumcised often find in the prepuce fibrous changes which favour the retention of smegma and a senile balanitis. Care and cleanliness are a sure preventive. In most elderly people the degenerative change in the bloodvessels favours the appearance of a mild œdema of the lower legs towards the end of the day. Grasping the legs in the hands and rubbing them firmly upwards to the knee at bedtime serves a double purpose. It passes the fluid into the circulation and secures a less limpid urine during the hours of sleep. It provides a less resistant area for the retirement of the blood from the brain to the lower limbs, which Howell recognises as an important physiological factor in the causation of sleep.

Some difference of opinion seems to exist among physiologists as to the normal contents of the rectum. In the older view it was a natural storehouse—more recently it has been thought to be usually empty with some storage near its upper end. Sir Arbuthnot Lane's researches give great prominence to the latter situation as one of frequent intestinal stasis, even when no prostatic enlargement is present. Dr. Hollis gives prominence to

"AS AGE CREEPS ON."—(*continued*).

the lower storage as the gerontal type, and advises the use of a protected finger to keep the prostate out of the way. The treacle enema—two tablespoonfuls of treacle with four of water—does not unduly distend the rectum and gives satisfactory relief. The higher storage is apt to lie back in the hollow of the sacrum and to become adherent to the wall of the gut. When the prostate is large, or the uterus retroverted, a semi-reclining, easy-chair posture at the beginning of defæcation keeps the obstructing mass out of the way and favours the expulsion of any adherent fæces. Cascara, and of the older fashion, aloes, have a special propulsive action upon this portion of the bowel, but they often fail to get rid of adherent fæces, and these may continue to give rise to irritation for some weeks. For such cases the yeast suppository—two introduced at bed-time and followed by a dose of cascara—is the most effectual method. When the block is low in the rectum half a teaspoonful of Parke, Davis and Co.'s cascara evacuant mixed with a teaspoonful of Heye's paste (confection of sulphur, senna, and black pepper in equal parts) is a convenient form. When the block is higher up, half a teaspoonful of the cascara evacuant and a teaspoonful of maltine with pepsin, pancreatin, and malt give a better result (Maltine Manufacturing Company). These measures are more or less preventive, but when the case is not seen in the early stage the bladder may have acquired the habit of not emptying itself completely. For such cases a tabloid of extract of the pituitary gland (Burroughs Wellcome and Co.) may be given once or twice a day for some time with good results.

There should be a regulation of foods as well as fluids. Breakfast should always be a substantial meal, with a fair allowance of fruits, avoiding those which contain much oxalic acid. Luncheon should be the meat meal of the day, with vegetables and followed by fruit, but with little or no fluid. Afternoon tea should have a good supply of fluid with little solid food. Dinner should consist chiefly of fish and chicken with vegetables, milk pudding, and very little fluid. Rectal relief is better sought on an empty stomach, before breakfast and before dinner, and always begun in the easy-chair posture. These directions read like a day's work, but when secured they free both day and night from much needless worry. The penalties which are inevitable consequences of advancing age are kept in abeyance and more grave consequences are averted. As habits, once acquired they become easy and lessen the burdens of advancing life in both sexes.

